# Unravelling Sex Disparities in the Pathophysiology of Atrial Fibrillation: Review of the Current Evidence

**DOI:** 10.1111/jce.70063

**Published:** 2025-08-18

**Authors:** Ibrahim Antoun, Georgia R. Layton, Ahmed Abdelrazik, Mahmoud Eldesouky, Hayley Davies, Osama Barakat, Amal Mahfoud, Abdulmalik Koya, Edward Y. M. Lau, Mustafa Zakkar, G. André Ng, Riyaz Somani

**Affiliations:** ^1^ Department of Cardiology University Hospitals of Leicester NHS Trust, Glenfield Hospital Leicester UK; ^2^ Department of Cardiovascular Sciences, Clinical Science Wing University of Leicester, Glenfield Hospital Leicester UK; ^3^ Department of Cardiac Surgery University Hospitals of Leicester NHS Trust, Glenfield Hospital Leicester UK; ^4^ Leicester British Heart Foundation Centre of Research Excellence Glenfield Hospital Leicester UK; ^5^ Department of Haematology University Hospitals of Leicester NHS Trust, Leicester Royal Infirmary Leicester UK; ^6^ Department of Medicine University of Aleppo Aleppo Syria; ^7^ Department of Medicine University of Latakia Latakia Syria; ^8^ National Institute for Health Research Leicester Research Biomedical Centre Leicester UK

**Keywords:** atrial fibrillation, gender, pathophysiology, remodelling, risk factors, sex

## Abstract

Atrial fibrillation (AF) is the most common sustained arrhythmia associated with increased risks of stroke, heart failure, and mortality. Men experience AF more frequently than women, but women are more likely to suffer greater symptoms and reduced quality of life as a consequence of AF onset. Its pathophysiology is complex, influenced by hormonal, structural, electrophysiological, and genetic factors. Sex hormones, including oestrogen, progesterone, and testosterone, play critical roles in modulating cardiac electrophysiology, autonomic function, and atrial remodelling, contributing to sex‐specific differences in AF prevalence and outcomes. Women experience increased AF risk post‐menopause due to declining oestrogen levels, while testosterone fluctuations in men are associated with arrhythmogenesis. Thyroid hormones further complicate the hormonal landscape by influencing cardiac excitability and autonomic regulation. Electrophysiological and structural differences between sexes, such as longer P‐wave durations and greater fibrosis in women, result in increased AF recurrence and complications, particularly after catheter ablation. Men, however, have a higher overall AF incidence, likely due to larger atrial sizes and different conduction properties. Lifestyle and psychological factors, including obesity, physical activity, and mental health, intersect with these sex‐specific risks, further influencing AF susceptibility. Artificial intelligence (AI) offers transformative opportunities to integrate these factors into personalised prevention and treatment strategies, enhancing early detection and tailored interventions. This review highlights the critical role of hormonal and sex‐specific factors in AF pathophysiology, emphasising the need for sex‐specific approaches to optimise management. Understanding these mechanisms is essential for developing targeted, personalised strategies to improve outcomes for men and women with AF.

## 1 Introduction

Atrial fibrillation (AF) is the most common sustained arrhythmia worldwide, posing significant public health and clinical challenges due to its associated risks of stroke, heart failure, and mortality [1, 2, 3]. AF management can be challenging, especially in the developing world, which has limited resources [4, 5, 6, 7, 8, 9, 10]. The underlying mechanisms of AF are highly complex and multifactorial, involving structural, genetic, and electrophysiological changes in the atria. Among these factors, hormonal influences have emerged as critical contributors to the pathophysiology and incidence of AF, particularly about sex‐specific differences.

Sex hormones, including oestrogen, progesterone, and testosterone, play a pivotal role in modulating cardiac electrophysiology, autonomic nervous system activity, and atrial remodelling [11, 12]. Cardiac myocytes express receptors for these sex hormones, and their activation modulates ion channel acidity to alter the electrical activity within the heart, suggesting that exposure to sex hormones can influence susceptibility to atrial fibrillation. One hypothesis is that sex hormones may modulate the effective refractory period (ERP), with observed differences in the recorded ERP within premenopausal women compared to post‐menopausal women and men [13] These hormonal influences are evident across the lifespan, with distinct patterns of AF prevalence and presentation between men and women. For instance, women tend to develop AF later in life, often after menopause, when declining oestrogen levels increase susceptibility to atrial remodelling and arrhythmogenesis. Studies have shown that the incidence of AF in women is 1.6 cases per 1000 person‐years, and in men, it is 3.8 cases per 1000 person‐years, with similar performance in European and Asian populations [14, 15, 16]. Additionally, thyroid hormones complicate the hormonal landscape by influencing cardiac excitability and autonomic function, highlighting the interplay between endocrine and cardiovascular systems in AF development.

Beyond hormonal effects, sex‐specific differences in atrial structure and electrophysiology, including disparities in P‐wave characteristics, atrial conduction, and fibrotic remodelling [17], have been well‐documented. These differences contribute to variations in AF susceptibility and influence treatment outcomes, such as catheter ablation success rates [18]. Lifestyle factors, psychological well‐being, and comorbid conditions further intersect with these sex‐specific mechanisms, underscoring the need for a comprehensive approach to AF management.

Despite advancements in understanding the role of sex hormones and sex disparities in AF, significant knowledge gaps remain. This article aims to provide an in‐depth analysis of the hormonal and sex‐specific factors influencing AF pathophysiology and incidence and their implications for prevention, diagnosis, and treatment. By exploring these mechanisms, the article highlights opportunities for personalised, sex‐specific strategies to optimise AF management and improve patient outcomes.

## 2 Hormonal Influences on AF Pathophysiology and Incidence

The pathophysiology of AF is multifaceted, with sex hormones playing a significant role in its incidence and progression. Understanding the hormonal influences on AF can provide insights into sex disparities in AF prevalence and outcomes, particularly among postmenopausal women and those with thyroid dysfunction. Many hormones throughout the body exert significant pathophysiologic effects on atrial tissue, thereby contributing to both structural remodelling and arrhythmogenesis.

The primary mechanisms by which these hormones result in enhanced AF susceptibility are elevated atrial fibrosis, ion channel function disruption, and autonomic nervous system activation and activation of inflammatory pathways, primarily by enhancing transforming growth factor‐beta (TGF‐β) signalling. Sex differences in hormonal secretion or action are complex, and many of the hormones essential for daily homeostasis do not necessarily demonstrate any clear gender‐specific differences. However, diseases directly influencing hormone secretion often affect differing patient phenotypes and have a propensity for one gender.

Catecholamines, such as epinephrine and norepinephrine, enhance automaticity and triggered activity via activation of β‐adrenergic receptors, shortening both the action potential duration and the refractory periods, thereby increasing susceptibility to AF [19]. Similarly, aldosterone promotes atrial fibrosis by stimulating fibroblast proliferation and extracellular matrix deposition, exacerbating potentially harmful electrical remodelling and inflammation [20].

Angiotensin II further drives atrial fibrosis through TGF‐β signalling and increases the heterogeneity of conduction, both of which elevate the risk of AF. Women, particularly premenopausal women, have lower angiotensin II activity due to oestrogen‐mediated suppression, reducing fibrosis and AF risk [21].

Thyroid hormones T3 and T4 amplify β‐adrenergic sensitivity, reducing action potential duration and refractory periods similar to catecholamines while simultaneously promoting atrial ectopy and re‐entry mechanisms. Thyroid hormones also play a crucial role in AF pathophysiology. Increased levels of thyroid hormones can lead to heightened sensitivity of the heart to catecholamines, resulting in shortened action potential duration and increased likelihood of AF [22]. This relationship underscores the importance of monitoring thyroid function in AF patients, particularly those with underlying thyroid disorders. The pro‐arrhythmic signalling associated with thyroid dysfunction further complicates the hormonal landscape influencing AF, necessitating a comprehensive approach to patient management that considers cardiac and endocrine health [23]. Thyroid disorders are some of the most common endocrinopathies in women before menopause. They are 3 to 5 times more common in women compared to men, and female sex is an independent risk factor for thyroid dysfunction, suggesting that these disorders, particularly if undiagnosed or untreated, may contribute to the enhanced AF morbidity in women, particularly in premenopausal women [24].

Atrial natriuretic peptide (ANP) and brain natriuretic peptide (BNP) are released in response to atrial stretch and exist to regulate volume homeostasis. However, in chronic overload states such as heart failure, they may also contribute to atrial remodelling. Persistent atrial stretch increases ANP and BNP secretion, which paradoxically activates profibrotic states; ANP modulates fibroblast activity, and chronic elevation of natriuretic peptides is associated with increased expression of TGF‐β and matrix metalloproteinases, leading to the collagen deposition resulting in atrial fibrosis. Natriuretic peptide levels are consistently elevated in women compared to men [25].

Cortisol also plays a role in the pathophysiology of AF, both directly and indirectly. Indirectly, it is associated with higher rates of hypertension and centripetal obesity, both known to be risk factors for AF [26]. Directly, it alters ion channel function and autonomic tone and promotes atrial fibrosis, increasing AF risk, particularly in chronic stress states. Its direct actions are exerted through stimulation of cardiac fibroblasts to laydown enhanced extracellular matrix collagens causing fibrosis, enhancing the profibrotic TGF‐ β pathways and increasing production of reactive oxygen species, thereby activating pro‐inflammatory cytokines such as tumour necrosis factor‐alpha (TNF‐α) and interleukin‐6 (IL‐6). Cortisol also induces sympathetic nervous system overactivity, thereby enhancing catecholamine release. Genetic studies have also identified an association between a genetic predisposition to higher cortisol and AF [27]. Compared to sex‐matched controls, women tend to have higher evening cortisol levels than men but lower levels earlier in the day. They also demonstrate lower stress reactivity profiles of cortisol release [28]. The sex‐specific cortisol profiles do not clearly explain how this may contribute to significant differences in AF risk.

The role of sex hormones in the pathophysiology of AF is, as expected, particularly noteworthy. The decline of oestrogen levels in postmenopausal women has been associated with an increased risk of AF. In contrast, lower testosterone levels in men have been linked to a higher AF incidence [29, 30].

Additionally, hormonal differences may contribute to cardiac structure and function variations, predisposing individuals to AF. Specifically, research has indicated that women may exhibit different patterns of atrial remodelling compared to men, potentially due to the influence of sex hormones on cardiac tissue [31, 32].

Sex hormones, particularly oestrogen and progesterone, have been shown to modulate the autonomic nervous system, which is known to influence AF occurrence. For instance, fluctuations in oestrogen levels throughout the menstrual cycle can affect cardiac electrophysiology, potentially leading to variations in AF symptoms and incidence among premenopausal women [33].

Given the complexity and multifactorial nature of any associations, the interplay of competing risks and the lack of high‐quality, observational or interventional study data, this modulation is largely hypothesised. It may be attributed to oestrogen's ability to enhance parasympathetic activity and reduce sympathetic tone, influencing heart rate and conduction properties. In postmenopausal women, the decline in oestrogen levels is associated with an increased risk of AF, as evidenced by studies indicating that women comprise a significant proportion of AF patients, particularly those aged 75 and older [34]. Moreover, the relationship between obesity, physical activity, and AF incidence in postmenopausal women highlights the interplay between hormonal changes and lifestyle factors.

Research indicates that obesity significantly increases the risk of AF, particularly in women, where hormonal changes post‐menopause may exacerbate this risk [34]. The interaction between obesity and physical activity suggests that lifestyle modifications could mitigate the risk of AF in this population, emphasising the importance of addressing both hormonal and behavioural factors in AF prevention strategies for all patients. In addition to oestrogen and thyroid hormones, androgens may influence AF incidence. Studies suggest that testosterone levels can impact cardiac function and arrhythmia susceptibility, with lower testosterone levels being associated with increased AF risk in men [32, 34, 35] (Figure 2). Decreased testosterone was linked to higher rates of body fat, hypertension, diabetes mellitus, and dyslipidemia, which leads to obesity and metabolic syndrome associated with atrial electric remodelling and AF development [36, 37, 38].

Furthermore, replacing testosterone in these patients reduced AF incidence [39]. This sex‐specific hormonal influence highlights the need for tailored approaches in AF management, recognising that hormonal profiles may differ significantly between men and women. The role of anxiety and depression in AF patients, particularly among women, cannot be overlooked. Psychological factors can exacerbate AF symptoms and may be influenced by hormonal changes, particularly during periods of hormonal fluctuation such as the menstrual cycle or menopause [40]. Addressing mental health in conjunction with hormonal and cardiovascular health may improve overall outcomes for patients with AF.

Furthermore, the structural changes associated with AF, such as fibrosis and atrial enlargement, are also influenced by hormonal factors. For instance, oestrogen has been shown to have protective effects against myocardial fibrosis, which significantly contributes to AF progression [41]. Understanding the interplay between hormonal influences and structural cardiac changes is essential for developing targeted therapies to prevent AF onset and progression. The increasing prevalence of AF, particularly among older women, necessitates a deeper understanding of the hormonal influences on its pathophysiology. This demographic shift underscores the urgency of addressing hormonal factors in AF research and clinical practice. Sex differences also play a role in the epidemiology of AF.

Collectively, many hormonal influences drive both structural and electrical changes in atrial tissue regardless of sex and diseases of the endocrine system can significantly predispose individuals to arrhythmias such as AF. The hormones that seem to have the greatest influence on AF risk in women compared to men are oestrogen and thyroid hormones.

## 3 Pregnancy and Hormonal Treatments

In general, premenopausal women have significantly lower rates of AF incidence than men; however, after menopause, especially after the age of 50, this gap diminishes, and the ratios between men and women become closer. This phenomenon suggests a protective role of oestrogen in women [16]. This is relevant in pregnancy, hormonal replacement therapy (HRT) and oral contraception (OC). The prevalence of AF was proposed to be 0.05% [42], usually occurring in women with heart diseases [43]. The hormonal cycle in women has also been recently studied, and the results suggest that early or late onset of menstruation and menopause, irregular cycles, and even reproductive years less than 30 years were all associated with increased risk factors and incidence of AF [44]. A variety of cardiovascular changes in normal women accompany pregnancy. At first, the levels of oestrogens increase and cause augmented adrenergic receptor sensitivity; also, the blood volume and the cardiac output increase; this results in the myocardial stretch and an increase in cardiac end‐diastolic volumes. Moreover, the augmented sinus heart rate may cause altered myocardial refractoriness, potentially setting up or stabilising re‐entry. All these changes in pregnant women are thought to promote arrhythmogenesis [45]. The increased pressure can promote atrial dilation and fibrosis, creating a substrate conducive to the development of AF [46]. For instance, women with rheumatic heart disease are particularly vulnerable, as the hemodynamic burden during pregnancy can exacerbate their condition, leading to AF and other complications [47, 48]. The incidence of AF in pregnancy is relatively low. Still, it can lead to serious complications, including heart failure and thromboembolic events, particularly in women with structural heart disease such as mitral stenosis [47]. Research has shown that the risk of AF increases with the number of pregnancies, suggesting a cumulative effect of pregnancy on cardiovascular health [49, 50, 51, 52]. The Women's Health Study highlighted a linear increase in AF risk with increasing parity, indicating that women who have had multiple pregnancies may be at greater risk for developing AF later in life [52]. Additionally, the increased heart rate and changes in autonomic tone during pregnancy can contribute to the development of AF. The sympathetic nervous system is often activated, which can lead to increased automaticity and ectopic foci in the atria, further predisposing women to arrhythmias [53, 54]. The presence of ectopic activity, which is common in healthy pregnant women, can also serve as a trigger for AF [55]. Given the unique physiological context of pregnancy, multidisciplinary management involving obstetricians, cardiologists, and anaesthetists is essential to optimise outcomes for both the mother and fetus. A comprehensive understanding of hormonal, structural, and hemodynamic factors influencing AF during pregnancy is vital for developing tailored treatment strategies in this population.

Hormone replacement therapy (HRT) and oral contraception (OC) have been a topic of considerable debate regarding their effects on cardiovascular health, particularly AF and thrombotic risk [56]. The influence of HRT and OCs, especially oestrogen therapy, on the risk of AF in women is multifaceted and has been the subject of various studies. Evidence suggests that while HRT can alleviate menopausal symptoms and reduce the risk of osteoporosis, it may also increase the risk of cardiovascular events, including AF and stroke [57, 58]. Research indicates that the use of HRT is associated with an increased risk of stroke in women with AF. A study by Apostolakis et al. found that women undergoing HRT resulted in a higher incidence of adverse outcomes, including ischemic stroke, compared to those not on HRT [57]. This is particularly concerning given that women with AF already face a higher risk of stroke than men, and the addition of HRT may exacerbate this risk [57]. The mechanisms underlying this increased risk may involve hormonal influences on vascular function and coagulation pathways, which can predispose individuals to thromboembolic events. Moreover, the timing of HRT initiation plays a critical role in its cardiovascular effects. Some studies suggest that starting HRT closer to the onset of menopause may have a more favourable risk profile compared to initiating therapy later [57]. This is consistent necessitating further research to clarify these relationships. In addition to oestrogen, the interplay between other hormonal factors and AF risk is noteworthy. For instance, fluctuations in progesterone and their potential effects on cardiac rhythm are areas of ongoing investigation [33]. The menstrual cycle's influence on AF symptoms has also been documented, suggesting that hormonal variations can affect the frequency and severity of AF episodes in premenopausal women [33]. This highlights the need for a nuanced understanding of how hormonal changes throughout a woman's life can impact AF risk and management.

## 4 Electrophysiological Differences in the Atria of Men and Women

Electrophysiological differences in the atria of men and women have been the subject of extensive research, particularly in the context of AF. Studies have shown that while there are some similarities in the electrophysiological characteristics of the atria between sexes, notable differences can influence AF's prevalence, presentation, and outcomes. One significant finding is that men and women exhibit differences in atrial conduction properties. Research indicates that men tend to have a higher success rate in cardioversion for atrial fibrillation compared to women, suggesting a potential sex‐based disparity in atrial electrophysiology [59]. This aligns with findings that men generally have larger right atrial volumes, which may influence conduction times and susceptibility to arrhythmias [60].

Furthermore, studies have shown that women often present with longer P‐wave durations and increased P‐wave dispersion, indicative of delayed atrial conduction and a higher risk for AF [61, 62]. These differences in P‐wave characteristics may reflect underlying structural and functional disparities in atrial tissue between sexes. Moreover, the remodelling of atrial tissue due to factors such as fibrosis also appears to differ by sex. Research has demonstrated that women with long‐standing persistent AF exhibit distinct patterns of fibrosis remodelling compared to their male counterparts, which can affect the electrical properties of the atria [31]. This remodelling is often linked to hormonal influences that differ between sexes, particularly the effects of oestrogen on cardiac tissue [63]. Such hormonal differences may contribute to the observed variations in atrial conduction and susceptibility to arrhythmias. In addition to structural differences, the electrophysiological response to interventions such as catheter ablation varies by sex. Women are often underrepresented in studies evaluating catheter ablation for AF. When they undergo the procedure, they may have different outcomes than men [64]. Women were proposed to exhibit more advanced atrial remodelling as assessed by high‐density electroanatomic mapping and experienced a higher recurrence of arrhythmias after AF ablation compared to men. These data call for a greater examination of facilitators and barriers to sustaining rhythm control strategies in women [65].

Outside of structural interventions, women also demonstrate enhanced complexity with drug therapy; they are more likely to experience side effects from antiarrhythmic drugs, primarily due to QT prolongation [66] Despite experiencing worse quality of life from AF symptoms and more drug‐linked complications, they are less likely to be referred for catheter ablation [67].

The CHA₂DS₂‐VASc score is widely used to assess stroke risk in patients with AF to support decision‐making regarding anticoagulation. Until recently, this system assigned an additional point for the female sex. However, the female sex was recently removed as an independent risk factor from the scoring because the impact of female sex on stroke risk is primarily seen only in women with additional risk factors for stroke and not in women without confounding risks [68]. This tool helps balance stroke prevention with bleeding risk when prescribing anticoagulants. The change in the scoring system acknowledges that the female sex is a risk modifier and also a prognostic indicator for stroke in patients with AF. Still, women without additional risk factors for stroke have very low baseline rates of stroke with AF [69].

**Figure 1:**
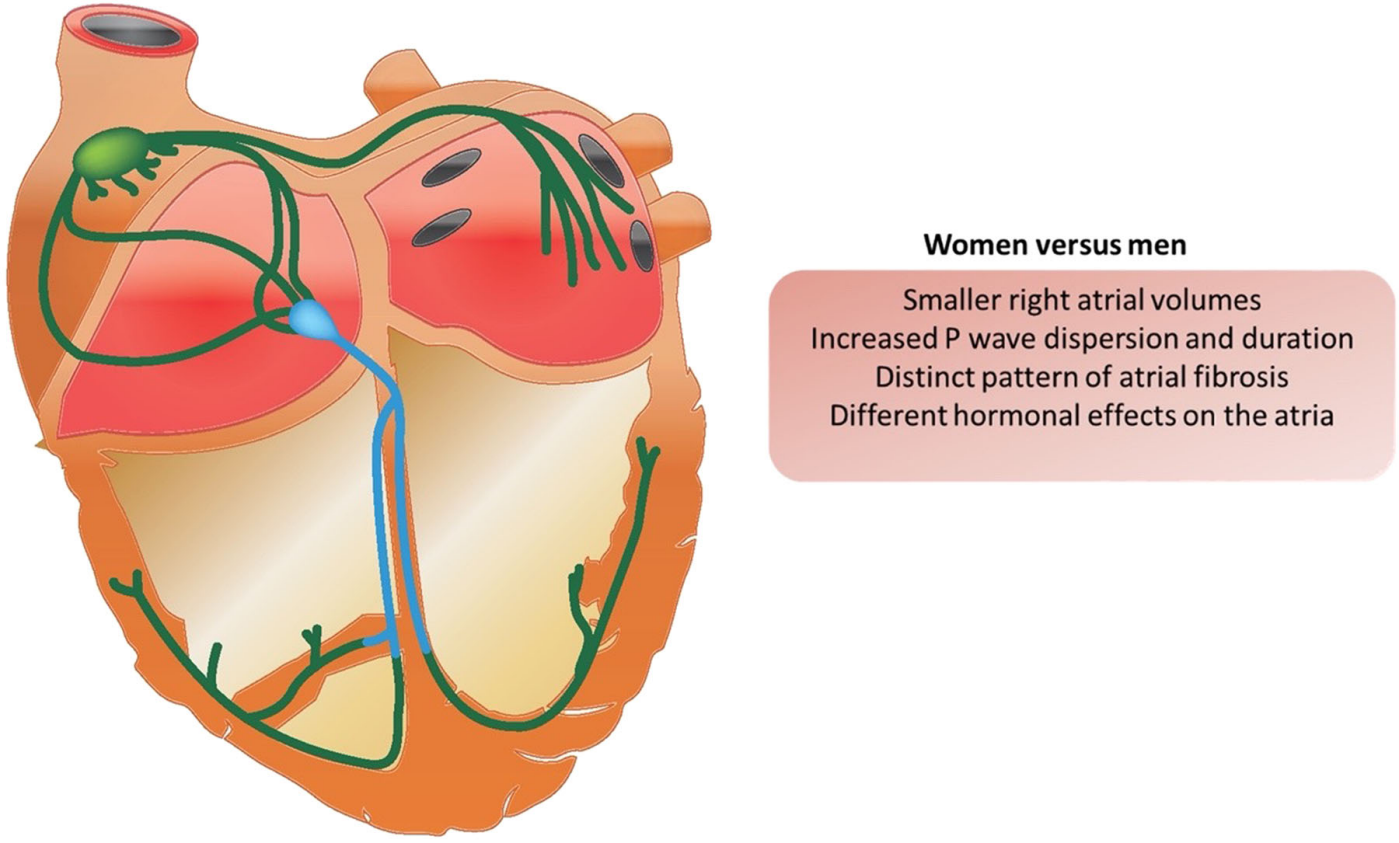
Electrophysiological differences in the atria of men and women.

These discrepancies in treatment choices and outcomes between sexes emphasise the need for sex‐specific approaches to managing AF, as the underlying electrophysiological mechanisms and morbidity profiles tend not to be matched across the sexes (Figure 1). Overall, while both men and women can experience AF, the electrophysiological characteristics of their atria differ significantly, influencing the clinical presentation and management of this common arrhythmia. Understanding these differences is crucial for tailoring treatment strategies and improving outcomes for both sexes.

**Figure 2:**
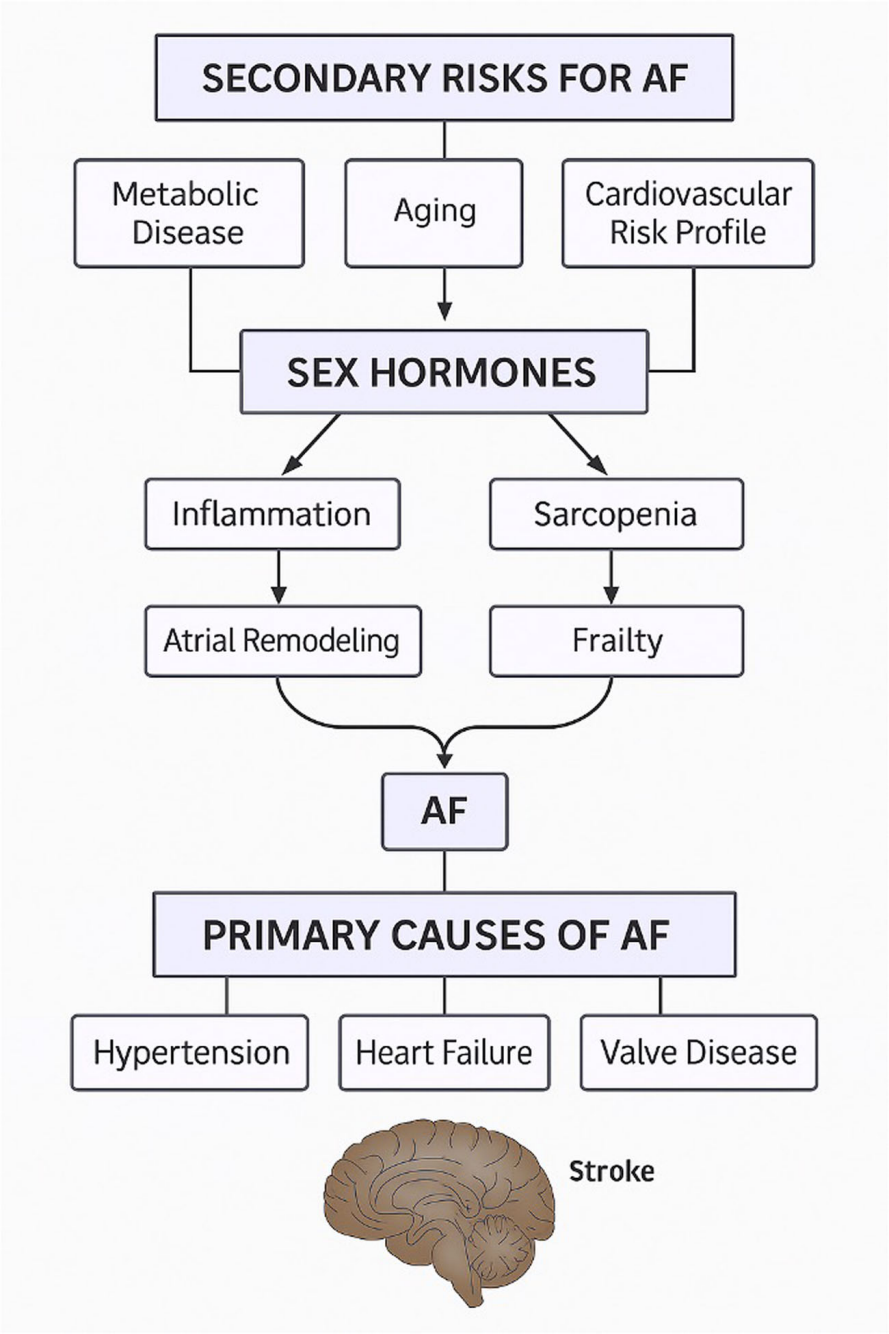
Schematic illustrating how metabolic, hormonal, and cardiovascular risk factors contribute to atrial fibrillation through sex hormone dysregulation and structural remodeling, alongside primary cardiac causes such as hypertension, heart failure, and valvular disease. AF, atrial fibrillation.

## 5 Sex Disparity in Atrial Remodelling

Sex disparity in atrial remodelling associated with AF is an increasingly recognised phenomenon in cardiovascular research. Studies have demonstrated that sex differences significantly influence the structural and electrical remodelling of the atria, affecting the clinical outcomes of AF patients. This response synthesises findings from various studies to elucidate the mechanisms and implications of these disparities. Research indicates that women with AF often exhibit distinct patterns of atrial remodelling compared to men. For instance, Marzak et al. found that female patients with persistent AF had lower voltage zones in the left atrium, indicative of more extensive atrial substrate remodelling [70]. Similarly, Wong et al. highlighted that women experience reduced conduction velocity and increased complex fractionated electrograms, contributing to higher recurrence rates of AF after catheter ablation [71]. These findings suggest that the electrical properties of the atria differ by sex, potentially due to hormonal influences and variations in atrial structure.

Moreover, Li et al. reported that women with long‐standing persistent AF have greater fibrosis remodelling than their male counterparts, which may be attributed to differential gene expression related to fibrotic processes [31]. This aligns with the observations of Westerman and Wenger, who noted that histological studies show increased atrial fibrosis in women with AF, a pattern not observed in men [49]. The implications of these findings are profound, as increased fibrosis is associated with poorer outcomes, including higher rates of stroke and systemic embolisation in women [72]. The role of sex hormones in atrial remodelling is also a critical factor. Studies have suggested that oestrogen may confer some protective effects against the development of AF, while its decline during menopause could exacerbate atrial remodelling and increase susceptibility to AF [72].

In contrast, men generally have a higher incidence of AF, which may be linked to their overall cardiovascular risk profile and the presence of comorbidities such as hypertension and diabetes [73, 74]. Additionally, the impact of lifestyle factors on atrial remodelling has been explored, with evidence suggesting that vigorous exercise may have differential effects based on sex. Benedetto et al. noted inconsistencies in the literature regarding physical activity's association with AF, indicating that while exercise is generally beneficial, excessive training could lead to pathological remodelling, particularly in men [75, 76]. Magnetic resonance imaging (MRI) studies suggest women have more fibrosis in their whole atria than men. Left atrial size is proportional to both body habitus and stature. For a given body and atria size, even if the sexes are matched, women may have more atrial fibrosis and be more primed for the development of AF [77].

In summary, the sex disparity in atrial remodelling associated with AF is characterised by distinct structural and electrical changes influenced by hormonal differences, genetic factors, and lifestyle choices. Women tend to exhibit more extensive atrial fibrosis and lower voltage zones, while men show a higher overall incidence of AF. Understanding these differences is crucial for tailoring treatment strategies and improving outcomes for both sexes.

## 6 Genetics and Molecular Mechanisms

Genetic factors also play a crucial role in the sex disparity observed in AF. Evidence suggests that certain genetic variants may be more prevalent in one sex compared to the other, influencing susceptibility to AF [78, 79].

Furthermore, familial patterns of AF have been documented, indicating a heritable component that may differ by sex [78]. There are five genes which have been highlighted through either genomic analysis or familial studies. These include TTN, MYL4, TBX5, NPPA, and KCNQ1. Variants within these genes are primarily associated with ion channel abnormalities (KCNQ1) [80], enhanced circulating levels of ANP (NPPA) [81] and atrial enlagrment (MYL4) [82]. Some of the most commonly identified variants known to be associated with AF, particularly early onset AF, have an as of yet unknown mechanism of action. For example, TTN is the most commonly identified gene where variant presence is associated with AF but how these variants contribute to AF onset is unknown [83, 84]. Identification of genetic variants associated with AF is an emerging field with limited supporting evidence. Therefore, how these variants may differ between men and women is currently not known. In a newly published study by Tokioka et al. [85], polygenic risk scores (synonymous with a person's genetic risk to AF) improved prediction of AF in men more so than women. This suggests that genetic contributions to AF may be less significant to female patients and that the differences in AF risk in women are more strongly associated with hormonal or environment factors. This also suggests that certain tools for AF screening and prediction need to be tailored by patient sex.

## 7 Sex‐Specific Risk Factors

The understanding of sex‐specific risk factors for AF is crucial for tailoring prevention and treatment strategies. Research indicates that various risk factors exhibit significant differences between sexes, influencing the incidence and outcomes of AF. One of the most prominent risk factors is age, a well‐established contributor to AF development. Studies have shown that men generally have a higher prevalence of AF at younger ages compared to women, who tend to develop AF later in life, particularly after menopause [86, 87]. This shift is attributed to hormonal changes, particularly the decline in oestrogen levels in postmenopausal women, which has been linked to increased susceptibility to AF [87, 88].

Additionally, obesity and hypertension are significant risk factors for both sexes, but their impact may differ. For instance, a study found that excess body mass index (BMI) and lower total cholesterol levels were more strongly associated with AF risk in men than women [89, 90]. Polycystic ovary syndrome is a novel risk factor for AF, which can be explained to hormonal changes and obesity [91]. Diabetes mellitus is another critical risk factor that exhibits sex‐specific effects. Women with diabetes have a higher relative risk of developing AF compared to their male counterparts, suggesting that diabetes may have a more pronounced impact on women [90, 92]. Furthermore, metabolic syndrome, which encompasses a cluster of conditions including hypertension, dyslipidaemia, and obesity, has been shown to increase AF risk, with some studies indicating that women may experience a greater burden from these conditions, especially in the developing world and the Middle East [93, 94].

Lifestyle factors also play a role in the sex disparity observed in AF. Smoking, for example, is a known risk factor for AF, and its prevalence varies between sexes with men overwhelmingly more likely to smoke than women [95], with more than a twofold increase in AF risk attributable to smoking in long‐term population studies [96]. Moreover, physical activity levels and adherence to healthy dietary patterns, such as the Mediterranean diet, have been associated with lower AF risk, with sex differences noted in adherence to these lifestyle modifications [97]. In terms of clinical presentation and outcomes, women with AF often present with more comorbid conditions, including heart failure and hypertension, which can complicate management and lead to worse outcomes compared to men [49]. This highlights the importance of considering sex‐specific factors in AF's clinical assessment and management. Therefore, the sex‐specific risk factors for atrial fibrillation encompass a range of biological, lifestyle, and clinical elements. Understanding these differences is essential for developing targeted prevention strategies and improving outcomes for both men and women affected by this arrhythmia.

In terms of clinical outcomes, women with AF often present with more comorbidities and a higher burden of symptoms, which can complicate management and treatment strategies [49]. Despite these challenges, women tend to have better long‐term survival rates compared to men, which may reflect differences in the underlying pathophysiology and response to treatment [16, 98]. This highlights the importance of considering sex‐specific factors in the management of AF, as therapeutic approaches may need to be tailored to account for these disparities.

## 8 Autonomic Nervous System Role Between Men and Women

The autonomic nervous system (ANS) plays a crucial role in the pathophysiology of AF, with its influence varying significantly between women and men. Understanding these differences is essential for developing sex‐specific treatment strategies and improving patient outcomes. The ANS comprises the sympathetic and parasympathetic nervous systems, which both regulate heart function and influence the initiation and maintenance of AF. Research indicates that the balance between sympathetic and parasympathetic activity is critical in the context of AF. For instance, sympathetic activation facilitates the onset of AF by increasing heart rate and enhancing atrial excitability.

In contrast, parasympathetic activation can have protective and pro‐arrhythmic effects depending on the context [99, 100]. In women, hormonal fluctuations throughout the menstrual cycle can modulate autonomic function, affecting the risk of AF. Studies have shown that parasympathetic activity is more dominant during the follicular phase, which may provide a protective effect against AF. Conversely, during the luteal phase, increased sympathetic activity may predispose women to arrhythmias [33, 101]. This cyclical variation in autonomic tone highlights the need for sex‐specific considerations in AF management. Moreover, the structural and functional differences in the heart between sexes can influence the autonomic regulation of cardiac rhythm. For example, women generally have smaller heart sizes and different atrial structures, which may affect how autonomic signals are processed and how they influence arrhythmogenesis [31].

Additionally, the presence of comorbidities, such as hypertension and heart failure, which often differ in prevalence and impact between sexes, can further complicate the autonomic regulation of AF [102, 103]. Recent studies have also indicated that the autonomic remodelling associated with AF can differ between men and women. For instance, women may experience more pronounced autonomic changes in response to AF, potentially leading to a greater burden of symptoms and complications. This suggests that interventions targeting the ANS, such as vagal nerve stimulation or pharmacological modulation, might need to be tailored according to sex to optimise their effectiveness. Therefore, the role of the autonomic nervous system in atrial fibrillation is complex and influenced by sex‐specific factors. Hormonal variations, structural differences in the heart, and comorbidities all contribute to how the ANS affects AF risk and management in men and women. Recognising these differences is vital for developing personalised treatment strategies to improve outcomes for both sexes.

## 9 Future Work

The integration of artificial intelligence (AI) into the management of AF presents a promising avenue for developing sex‐specific approaches to mitigate AF risk in both men and women. AI‐driven methodologies can enhance the detection, prediction, and personalised treatment of AF, thereby addressing the unique risk profiles associated with each sex. One key area where AI can contribute is in the early detection of AF. Machine learning algorithms, particularly those utilising ECG data, have shown significant potential in identifying AF and predicting its onset. For instance, studies have demonstrated that deep learning models can effectively classify AF from ECG signals, providing a noninvasive and efficient patient screening method. By tailoring these algorithms to account for sex‐specific risk factors, such as hormonal influences and comorbidities, healthcare providers can improve early detection rates and initiate preventive measures more effectively. Moreover, AI can facilitate personalised treatment strategies by analysing large datasets to identify patterns and correlations that may differ between the sexes. For example, a study highlighted using machine learning to predict AF recurrence after catheter ablation, a common treatment for AF. By incorporating sex‐specific data into these predictive models, clinicians can better tailor ablation strategies and post‐procedural care to the individual needs of male and female patients, potentially improving outcomes and reducing recurrence rates. Genetic factors also play a significant role in AF susceptibility, and AI can assist in identifying genetic biomarkers that may differ between sexes. Research has shown that certain genetic polymorphisms are associated with AF recurrence and response to treatment [104, 105]. By leveraging AI to analyse genetic data alongside clinical information, healthcare providers can develop more accurate risk stratification tools that consider genetic predispositions and sex‐specific factors, leading to more effective management strategies.

Furthermore, AI can enhance the management of comorbid conditions that often accompany AF, such as hypertension and diabetes, which also exhibit sex differences in prevalence and impact. For instance, machine learning models have been employed to predict the risk of new‐onset AF in patients with hypertension, allowing for proactive blood pressure management and other risk factors [106]. By focusing on these comorbidities through a sex‐specific lens, AI can help mitigate the overall risk of AF in both men and women.

## 10 Conclusion

AF is a complex arrhythmia influenced by numerous factors, including hormonal, genetic, structural, and electrophysiological differences that are specific to sex. Hormonal influences, particularly fluctuations in oestrogen, progesterone, thyroid hormones, and androgens, play a crucial role in the pathophysiology of AF, emphasising the need to address these factors in both prevention and treatment strategies. Women face unique risks due to hormonal fluctuations, a menopause‐related decline in oestrogen, and a greater susceptibility to fibrosis. In contrast, men often present with a higher overall incidence of AF linked to testosterone levels and cardiovascular comorbidities. Electrophysiological and structural differences, such as variations in atrial conduction and fibrosis remodelling, further highlight the necessity for sex‐specific approaches. For example, women exhibit shorter P‐wave durations and distinct fibrosis patterns, which are associated with increased recurrence and complications, particularly after interventions like catheter ablation [107]. Conversely, men demonstrate larger atrial sizes and varying voltage zones, which influence arrhythmogenesis and therapy response. The autonomic nervous system also mediates sex‐specific AF risks, with hormonal fluctuations modulating autonomic tone differently in men and women. These variations influence susceptibility to AF and underscore the importance of personalised treatment strategies targeting both autonomic and structural remodelling.

Future efforts must prioritise research into these sex‐specific mechanisms to optimise AF management. AI presents a transformative opportunity to incorporate these sex differences into diagnostic, predictive, and therapeutic tools. By leveraging AI, clinicians can enhance early detection, refine treatment approaches, and address the unique physiological and lifestyle factors predisposing men and women to AF.

In conclusion, sex‐specific factors significantly influence the pathophysiology, incidence, and outcomes of atrial fibrillation. A nuanced understanding of these differences is critical for advancing personalised, sex‐tailored prevention and treatment strategies and improving outcomes for all patients with AF.

## Author Contributions

**Ibrahim Antoun:** conceptualization, methodology, validation, writing original manuscript. **Georgia R. Layton:** writing original manuscript, writing – review and editing. **Abdulmalik Koya, Ahmed Abdelrazik, Mustafa Zakkar, G. André Ng, Amal Mahfoud, Mahmoud Eldesouky, Osama Barakat, Edward Y. M. Lau, G. André Ng,** and **Riyaz Somani:** writing – review and editing.

## Consent

The authors have nothing to report.

## Conflicts of Interest

The authors have no conflicts of interest.

## Data Availability

The data that support the findings of this study are available from the corresponding author upon reasonable request.
